# Proceedings of the Cook County Medical Society

**Published:** 1855-10

**Authors:** 


ARTICLE II.—*Proceedings of the Cook County Medical Society.* Large doses of Tinct. Opii., without any poisoning effects, &c.

THE Cook County Medical Society held its regular monthly meeting on the first Tuesday in September. A respectable number of members were present, and the meeting was both interesting and profitable. Edmund Andrews, A. M., M. D., was duly proposed and elected a permanent member of the Society. After the transaction of some other business of no general interest, the reading of reports and communications being called for, Dr. Paoli read a paper detailing an account of a case of labor and of monstrosity or imperfect fœtal development.

The mother was attacked, shortly before the commencement of labor, with a pretty copious uterine hemorrhage, for the suppression of which she took two *drachms* of oil of turpentine, and had cold water applied over the pubes.

The hemorrhage soon ceased, but labor-pains subsequently came on, and expelled a fœtus of six or seven months growth, but of very defective formation. Among other anomalies, the umbilical cord was detached from the fœtus and the intestines were protruding from an apparent rupture in the abdominal walls. The only vestige of umbilical cord which could be found was a piece about two inches long attached to the placenta. The placenta, itself was much diseased throughout its whole extent; and in some places appeared to have undergone partial decomposition.

Dr. P., said the fœtus had the appearance of having been dead from twenty-four to forty-eight hours before its expulsion, but he thought the morbid condition of the placenta of much longer duration. Dr. Bevan reported verbally a case in which an unusual quantity of tincture of opium was taken without any severe effects. The patient was a man aged about thirty years, of intemperate habits, and evidently laboring under partial insanity at the time. On the 29th of August he procured two ounces of Tinct. Opii. for the purpose of killing himself.

In the evening he took one ounce of it; during the night half an ounce more, and in the morning of the 30th, he took the remaining half ounce. After waiting until 10 o'clock, A. M., without any serious effects, he went to a Drug-store near by, and procured four ounces more of the laudanum. He took one half of this (℥ij) before 11 o'clock, A. M., and between three and four P. M., he took the remainder, except about ℥½, which was left in the vial; and was presented to the Society for examination.

A few minutes after he took the last dose, he seemed to realize his condition, and hastened directly to Dr Bevan's office, to whom he related what he had done. The doctor immediately administered an emetic, which operated freely. The matter ejected from the stomach, both in color and smell gave evidence of being strongly impregnated with laudanum. No marked sleepiness or other unpleasant symptoms followed, except a pain in the occipital region, and retention of urine. The latter made the use of the catheter necessary. On the first of September, he was able to patronize the dram-shops as usual, and became thoroughly intoxicated. There is, of course, no absolute certainty that the man took all the laudanum stated above; but the emptiness of his vials, and the fact (fully ascertained) that he had purchased the quantity named, all corroborated his own statement, and rendered it probable that he had actually taken the whole amount. Good judges pronounced the specimen of laudanum exhibited to be of good quality, and prepared in accordance with the directions of the United States Dispensatory.

Prof. A. B. Palmer, also related a case which came under his observation not long since. The patient was a Scotchman by birth, and had been much affected by domestic troubles, which he attempted to drown by drinking beer. Not succeeding in this he attempted to kill himself with laudanum. For this purpose he purchased two ounces of laudanum and nearly a pint of whiskey; took them to his room, and in the evening, drank about half of the whisky and *all* the laudanum. He also took the same evening an ounce of a mixture of Tinct. Hyosciamus, Tinct. Opii. and Tinct. of Camphor about equal parts, and went to bed. The next morning he was found in bed sleeping rather soundly, with face purplish or of a livid color. A physician was called, who attempted to give an emetic. But the patient aroused up and positively refused to take any medicine, saying that he knew what he wanted. Dr. Palmer saw him in the afternoon.

The pupil of the eye was strongly contracted, and the patient sleepy though easily roused. He drank freely of tea and coffee, and recovered without any vomiting or dangerous symptoms. He had retention of urine, however, and symptoms of delirium tremens for two or three days.

The question how far the co-incident use of alcoholic drinks and incipient insanity or delirium is capable of counteracting the effects of opium on the system, was discussed by members of the Society, but elicited no additional facts of importance.

The following case recently sent us by Dr. Dodson, has a bearing on the same question, and on that account, may be of interest to the reader.

